# Computational Investigation of Cerebrospinal Fluid Dynamics in the Posterior Cranial Fossa and Cervical Subarachnoid Space in Patients with Chiari I Malformation

**DOI:** 10.1371/journal.pone.0162938

**Published:** 2016-10-11

**Authors:** Karen-Helene Støverud, Hans Petter Langtangen, Geir Andre Ringstad, Per Kristian Eide, Kent-Andre Mardal

**Affiliations:** 1 Center for Biomedical Computing, Simula Research Laboratory and Department of Informatics, University of Oslo, Oslo, Norway; 2 Department of Radiology and Nuclear Medicine, Oslo University Hospital- Rikshospitalet, University of Oslo, Oslo, Norway; 3 Department of Neurosurgery, Oslo University Hospital- Rikshospitalet, Faculty of Medicine, University of Oslo, Oslo, Norway; 4 Department of Mathematics, University of Oslo, Oslo, Norway; University of Washington, UNITED STATES

## Abstract

**Purpose:**

Previous computational fluid dynamics (CFD) studies have demonstrated that the Chiari malformation is associated with abnormal cerebrospinal fluid (CSF) flow in the cervical part of the subarachnoid space (SAS), but the flow in the SAS of the posterior cranial fossa has received little attention. This study extends previous modelling efforts by including the cerebellomedullary cistern, pontine cistern, and 4th ventricle in addition to the cervical subarachnoid space.

**Methods:**

The study included one healthy control, Con1, and two patients with Chiari I malformation, P1 and P2. Meshes were constructed by segmenting images obtained from T2-weighted turbo spin-echo sequences. CFD simulations were performed with a previously verified and validated code. Patient-specific flow conditions in the aqueduct and the cervical SAS were used. Two patients with the Chiari malformation and one control were modelled.

**Results:**

The results demonstrated increased maximal flow velocities in the Chiari patients, ranging from factor 5 in P1 to 14.8 in P2, when compared to Con1 at the level of Foramen Magnum (FM). Maximal velocities in the cervical SAS varied by a factor 2.3, while the maximal flow in the aqueduct varied by a factor 3.5. The pressure drop from the pontine cistern to the cervical SAS was similar in Con1 and P1, but a factor two higher in P2. The pressure drop between the aqueduct and the cervical SAS varied by a factor 9.4 where P1 was the one with the lowest pressure jump and P2 and Con1 differed only by a factor 1.6.

**Conclusion:**

This pilot study demonstrates that including the posterior cranial fossa is feasible and suggests that previously found flow differences between Chiari I patients and healthy individuals in the cervical SAS may be present also in the SAS of the posterior cranial fossa.

## Introduction

In Chiari I malformation, the cerebellar tonsils are by definition at least 3–5 mm below the level of the foramen magnum (FM). The condition is typically diagnosed on static magnetic resonance images (MRI). Symptoms range from severe headache to sleep apnea and muscle weakness. Unfortunately, there is no direct correlation between symptoms in Chiari patients and to which extent the cerebellar tonsils are displaced, which motivates research on flow dynamics [1].

The Chiari I malformation is associated with abnormal cerebrospinal fluid (CSF) flow. Phase contrast MRI (PC-MRI) of patients with Chiari I malformation demonstrates abnormal flow in the FM and upper spinal canal such as increased peak velocities, bidirectional flow, flow jets and phase differences between peak velocities [2]. Abnormal CSF velocities suggest abnormal pressure gradients, which has been suggested as the underlying cause of syringomyelia development [2] that might accompany the Chiari I malformation. Recently, it was reported abnormal intracranial-lumbar pulsatile intracranial pressure (ICP) gradient in 7/10 Chiari I patients [3]. However, there is no clear association between tonsillar ectopy and scores of static or pulsatile ICP [3,4].

Computational fluid dynamics (CFD) provides both pressure and velocity parameters with high temporal and spatial resolution, and has consequently become a popular tool for simulating the flow changes caused by the Chiari I malformation [5–9]. Both patient specific and idealized geometries have been used in CFD studies. It has been suggested that flow resistance and flow impedance potentially provide more precise measures of the severity of the subarachnoid space (SAS) obstruction and may be quantified accurately by CFD [10]. Hence, CFD models have clearly improved the understanding of CSF hydrodynamics. Most previous CFD studies have cut the geometry at or just above the FM to reduce the complexity of the computer model. To our knowledge, only the model by Gupta et al. [11] includes the cerebellomedullary cistern, pontine cistern, and the 4th ventricle. However, they only considered flow down to the level of C1 in the normal SAS. The aim of the present work was to include the posterior cranial fossa CSF flow by incorporating the cerebellomedullary cistern, pontine cistern, and 4^th^ ventricle in order to compare variations in both posterior cranial fossa and cervical CSF flow. We obtained patient-specific PC-MR flow conditions in the aqueduct and cervical subarachnoid space. To quantify CSF flow variations we compared velocity patterns, pressure drop, and flow resistance in the cervical and posterior cranial fossa SAS in two Chiari patients and one control subject with normal anatomy.

## Methods

### Individuals/subjects

The Norwegian South-East Regional Ethics Committee (S-07237) and Institutional Review Board (07/5869) approved the study. All participants provided written informed consent.

The current study included three individuals/subjects:

- A 40 years old female control with normal craniospinal anatomy, hereafter denoted Con1.
- A female 26 years old female patient with Chiari I malformation and a distinct syrinx at the level of C2, hereafter denoted P1. She had for 2–3 years experienced headache, fatigue and dizziness, with impaired work-capability.
- A female 63 years old patient with Chiari I malformation, hereafter denoted P2. She had suffered from dizziness, and some unsteady gait, diplopia and dysphagia about 2 years prior to assessment.

Following assessment, both P1 and P2 underwent neurosurgery with occipital decompression and later permanent cerebrospinal fluid (CSF) diversion by shunt surgery. Both responded well to surgery with good outcome and improvement of pre-operative symptoms.

### MRI

The MR images was obtained at Oslo University Hospital on a 3T Siemens scanner (Skyra) for Con1, and on a 3T Philips scanner (Achieva 2.5.3) for P1 and P2. For the segmentation of the SAS and ventricles for the CFD simulations, we used T2-, heavily fluid weighted 3D steady state echo images with a spacing of 0.5 mm x 0.5 mm or 1 mm x 1 mm in the sagittal plane and slice thickness of 1 mm.

PC-MRI was obtained on both scanners at the same levels below the FM and at the aqueduct. At the aqueduct, the velocity encoding (V_enc_) was set to 8 cm/s for P1 and P2, while it was set to 16 cm/s for Con1. At the level of FM, the V_enc_ was set to 6 cm/s for P1 and P2 and 10 cm/s for Con1. The spatial resolution was 0.62 mm x 0.62 mm, slice thickness was 4.0 mm, and 32 images were obtained per cardiac cycle using retrospective cardiac gating.

### Construction of patient specific geometries from MRI

**Fig 1:**
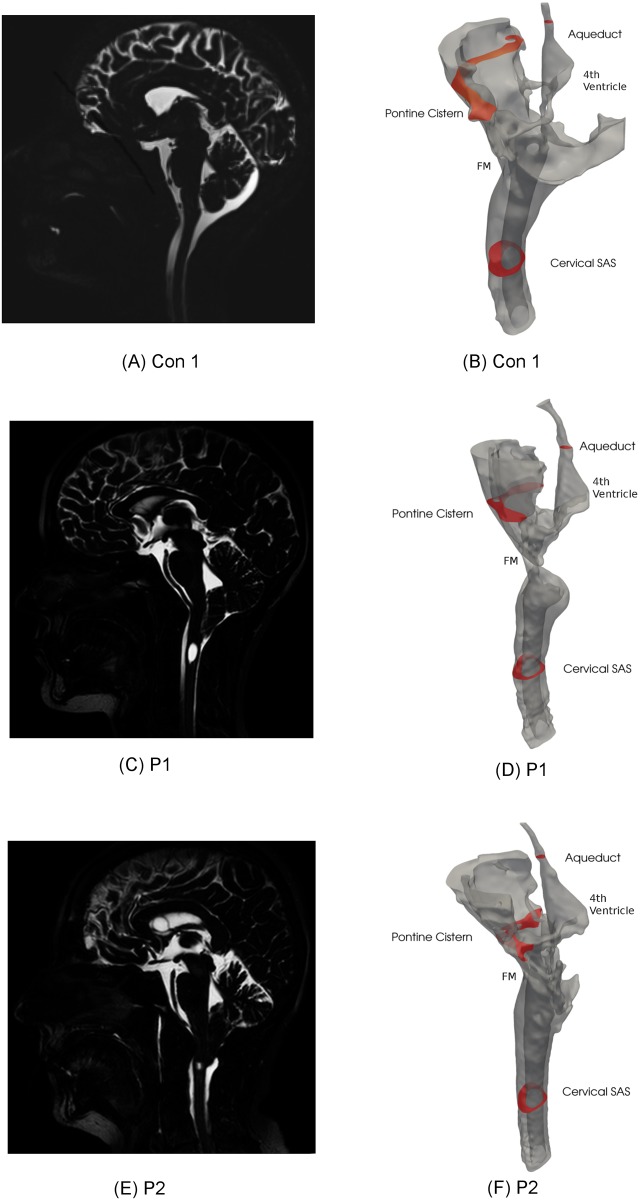
Sagittal MRIs and corresponding level set surfaces. Sagittal MRIs of Con1 (A), P1 (C), and P2 (E). Corresponding surface meshes are displayed in (B), (D), and (F), where the selected cross-sections at which pressure is evaluated is indicated in red.

The anatomy of the cervical subarachnoid space, pontine cistern, and the 4th ventricle including the aqueduct, was reconstructed using the Vascular Model Tool Kit (VMTK) [12]. The segmentation algorithms in VMTK are based on level set methods, which were used to extract surfaces representing the dura mater and the pia mater or brain tissue. Care was taken to make the surface true to the underlying image and surface smoothing was kept at a minimum. The extracted surfaces were smoothed and opened in each end to create inflow and outflow boundaries. Finally, computational meshes were generated with VMTK for Con1, P1, and P2. In Fig 1 midsagittal MR images and corresponding segmented level set mesh surfaces are displayed.

### Computational Fluid Dynamics

To simulate CSF flow through the spinal SAS, we employed the Navier-Stokes equation for an incompressible Newtonian fluid:

$$\begin{array}{r} \frac{\partial u}{\partial t}+u\cdot \nabla u-\nabla \cdot \sigma (u,p)=0,\\ \nabla \cdot u=0. \end{array}$$ (1)

Here, **σ** is the Cauchy stress tensor, which is dependent on the strain tensor **ε** and for a Newtonian fluid **σ** is given by

$$\begin{array}{c} \sigma (u,p)=2\nu \epsilon (u)-pI,\\ \epsilon (u)=\frac{1}{2}\left(\nabla u+\nabla^{T}u\right). \end{array}$$

The primary variables, **u** and *p*, describe the unknown CSF velocity and pressure, respectively. The stress (**σ**) is dependent of the kinematic viscosity ν = μ/ρ, which involve the fluid density ρ and the dynamic viscosity μ, as well as the pressure and velocity gradient. CSF is, as mentioned, a water-like fluid and behaves as a Newtonian fluid with ρ and μ similar to water at body temperature. Thus, in all simulations we used ρ = 1000 [m/kg^3^] and μ = 7.0 · 10^−4^ [kgs/m]. To solve the governing equations we employed a semi—implicit incremental pressure correction scheme (IPCS) [13] implemented in a previously verified and validated simulator that is an extension of the solver* used in Støverud et al (2013) [14] based on the FEniCS software framework [15].

A consequence of any pressure correction scheme is that boundary conditions for both velocity and pressure must be assigned on the entire boundary (either explicitly or implicitly). In this study, we used homogenous Neumann boundary conditions ∂*p*/∂**n = 0** on all boundaries combined with Dirichlet conditions for the velocity, as described in detail on page 9. For a more thorough discussion of appropriate boundary conditions in the present case, see Langtangen et al. [13].

### Patient specific boundary conditions

The PC-MRI data was analysed in BioFlows (tidam.fr). Based on spectral segmentation, a region of interest (ROI) was chosen, i.e., the region with CSF flow. Then, volume flux and velocity curves as a function of time were automatically calculated and saved in a spreadsheet. The heart rates were 71, 86, and 77 beats per minute for Con1, P1, and P2, respectively.

The quality of the measured data was high for P1. For P2, the V_enc_ was set too low and an antialiasing filter was applied to achieve flow in a uniform direction at each time step. For Con1, the V_enc_ value was set to 10 cm/s at FM, which was high and caused a low signal to noise ratio and thus uncertainties in the data. Therefore, part of the ROI was determined manually in Con1. The data, ROIs and filtrations were analysed together with a radiologist (GAR) and found adequate for the study. In 2D PC-MRI only the velocity component perpendicular to the selected slice is measured and in the simulations we therefore assumed the flow to be perpendicular to the slices.

**Fig 2:**
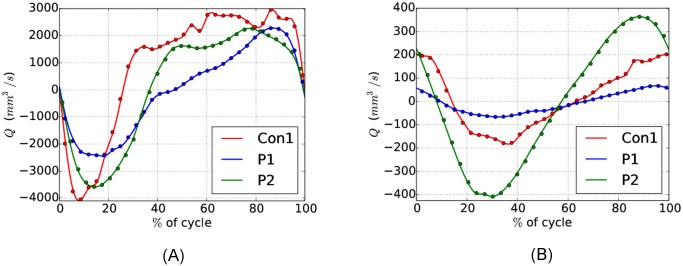
Measured volume flux through the SAS at the level of C1 (A) and in the aqueduct (B).

The resolution of the data is relatively low both in time and space compared to the resolution in the simulations. To be able to evaluate the boundary conditions at any point in time, we interpolated between the measured points using cubic splines. The computed flux and corresponding splines can be seen in Fig 2. Negative velocities correspond to caudal flow.

To compute a pointwise velocity $u_{\Gamma_{i}}$, at the inflow/outflow boundaries Γ_i_, we distributed the volume flux over the boundary according to a weighting function τ(**x**). This velocity was set to zero at the wall and increased linearly with the distance from the wall. To be specific, the velocity profile is

$$u_{\Gamma_{i}}=\frac{Q_{i}(t)\tau (x)}{\int_{\Gamma_{i}}\tau (x)d\Gamma_{i}}n$$ (2)

The above inflow/outflow conditions were applied at the caudal end and at the aqueduct. Flow was not measured in the pontine or cerebellomedullary cisterns, and therefore the principle of mass conservation was used to construct velocity profiles. The cerebellomedullary cistern was only present in the control. That is, we subtracted the flux through the aqueduct from the flux at the FM, and the remaining flux was divided into the two areas and scaled by the areas. A no-slip boundary condition **u** = 0 was assumed at the SAS boundaries.

### Computations

Simulations were performed in all three segmented geometries. The simulations were performed with a time step of Δt = 10^−4^ s for three cardiac cycles. Simulations were initiated with zero-flow velocity, but a steady periodic state was reached already in the second flow cycle.

For the velocity, we evaluated flow patterns at peak systolic inflow, while for the pressure, we computed the spatial average as a function of time at three selected slices in the cervical SAS (CS), aqueduct (Aq), and pontine cistern (PC), as indicated by the red slices in Fig 1. The slice at CS is taken at C2 and as a reference point in the images they are taken at height 35 mm. Corresponding slice in PC is at 110 mm and in the Aq the slices are at 135 mm in P1 and P2, and at 128 mm in Con1 (which was very narrow at 135 mm). Then we defined the pressure drop between PC and CS as

$$\Delta p_{PC-CS}=p_{\text{PC}}-p_{\text{CS}},$$ (3)

and the pressure drop between Aq and PC as

$$\Delta p_{Aq-CS}=p_{\text{PC}}-p_{\text{CS}}.$$ (4)

### Hydrodynamical measures

Flow resistance has previously been considered as a measure of obstruction in Chiari patients [10,14]. Here, the following was computed: resistance between PC and CS, defined as

$$R_{PC-CS}=\frac{\Delta p_{{\left(PC-CS\right)}_{\text{max}}}}{Q_{CS_{\text{max}}}},$$ (5)

where $Q_{CS_{\text{max}}}$ is the peak systolic flow at the cervical inlet, and the resistance between Aq and PC was defined as

$$R_{Aq-PC}=\frac{\Delta p_{{\left(Aq-PC\right)}_{\text{max}}}}{Q_{{\text{Aq}}_{\text{max}}}},$$ (6)

where $Q_{Aq_{\text{max}}}$ is the peak systolic flow in the aqueduct. Furthermore, the phase difference θ between Δp and **u** was computed as

$$\theta =\frac{t_{u_{\text{max}}}-t_{\Delta p_{{\left(PC-CS\right)}_{\text{max}}}}}{T},$$ (7)

where $t_{Q_{\text{max}}}$ represents the time of peak systolic velocities in CS, $t_{\Delta p_{{\left(PC-CS\right)}_{\text{max}}}}$ the time of peak differential pressure, and *T* the duration of caudal flow during the time of systole.

### Sensitivity to spatial mesh resolution

**Fig 3:**
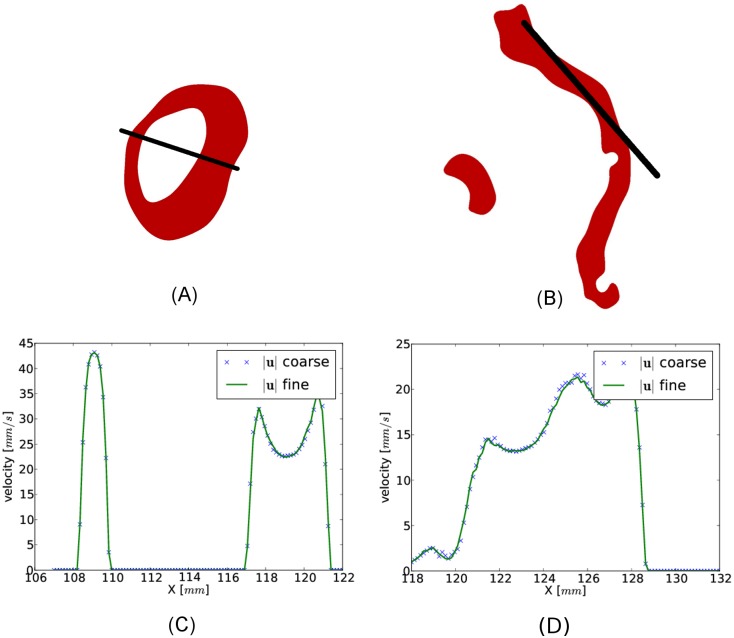
Comparison of velocity in a fine and coarse mesh. Plot of the magnitude of the velocity (|**u**|) at peak systolic flow along the lines indicated in (A) and (B) for the coarse and the fine mesh in Con1 (see Fig 2B). The maximum difference in the velocities in the chosen sections was approximately 10%.

To check the results for mesh independence, all simulations were performed on two meshes with different resolutions. The meshes corresponding to Con1 had 9.95 and 18.5 million cells, for P1 the meshes had 8.02 and 16.8 million cells, and for P2 they had 7.91 and 18.9 million cells. We selected two slices where we compared the magnitude of the velocity (|**u**|) along the lines indicated in Fig 3. We defined the maximum percentage difference between the velocities in the two meshes as

$$e_{\left|u\right|}=100\%\cdot \text{max}\left|\frac{\left|u_{\text{min}}^{coarse}|-|u_{\text{min}}^{\text{fine}}\right|}{\left|u_{\text{min}}^{\text{fine}}\right|}\right|,$$ (8)

where |**u**|_max_ represents the magnitude of the peak velocity. Note that negative velocities are in the caudal direction and positive velocities in the cranial direction. For the pressure, we similarly defined the maximum difference between the computations in the two meshes as

$$e_{\Delta p_{PC-CS}}=100\%\cdot \frac{\Delta p_{{\left(PC-CS\right)}_{\text{max}}}^{\text{coarse}}-\Delta p_{{\left(PC-CS\right)}_{\text{max}}}^{\text{fine}}}{\Delta p_{{\left(PC-CS\right)}_{\text{max}}}^{\text{fine}}}$$ (9)

where $\Delta p_{{\left(PC-CS\right)}_{max}}$ s the maximum difference in pressure over the whole cycle when comparing pressure averaged at the selected slices in the pontine cistern and cervical SAS.

## Results

### Mesh independence

We have plotted the magnitude of peak systolic velocity along the lines in the selected slices in PC (110 mm from bottom of the image) and CS (35 mm) for the coarse and fine mesh in Con1 Fig 3. The resulting curves for P1 and P2 were similar and are thus not displayed. At peak systolic flow, the maximum discrepancy defined in Eq 8 was less or equal to 10% in all subjects outside the vortices. Due to low velocities close to the wall, the relative difference measure was in some cases larger than 10% here. The differential pressure $\Delta p_{{\left(PC-CS\right)}_{max}}$ varied by less than 1% between the two meshes for all three subjects. In an accompanying study [16] we have resolved the flow in full detail, i.e., performed a direct numerical simulation and demonstrated that cell sizes down to 0,01 mm was needed to resolve the flow. Such high resolution was not possible with the computational methods used in this study. Pressure drop and maximal velocities were however similar.

### Velocity

**Fig 4:**
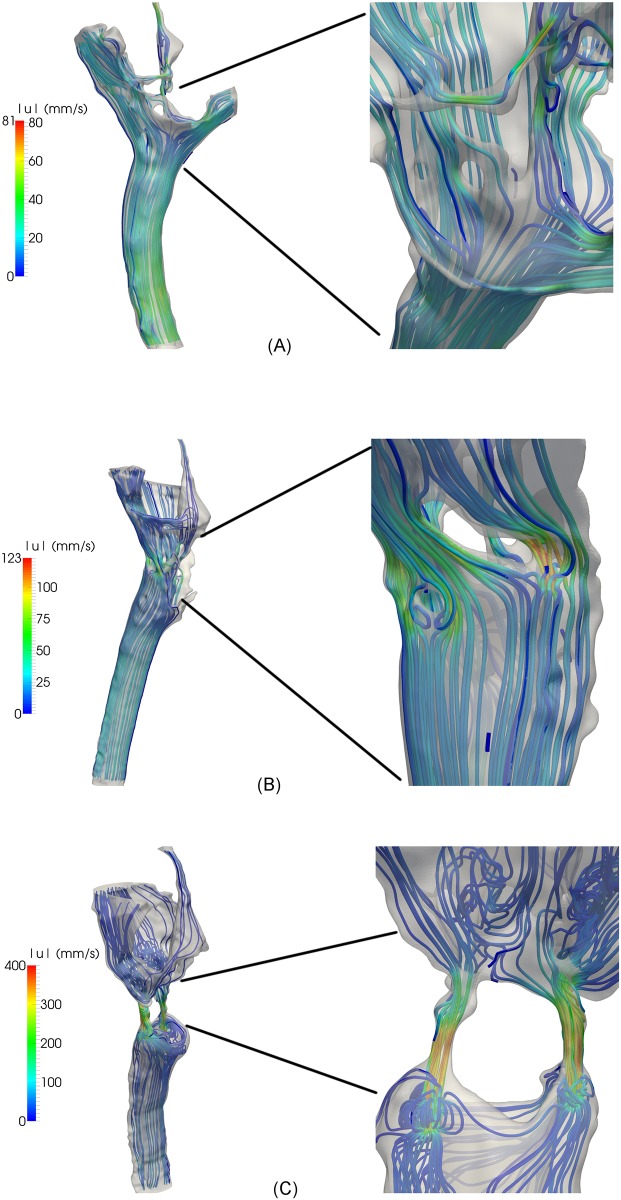
Streamlines at the time of peak systolic inflow in Con1 (A), P1 (B) and P2 (C). (A) In Con1 the flow is uniform and unidirectional. In the cervical SAS, the velocities increased with distance from FM. Zooming in on flow details in (A) reveals that there was a tendency to vortex formation in the fourth ventricle because of flow in opposite directions. (B) In P1 the maximum velocities were higher than Con1, even though the peak inflow was lower. Figure (B) shows a jet in the narrow passage between the intracranial and cervical SAS and a tendency to vortex formation. (C) In P2 the velocities were almost four times higher than in P1. Zooming in on details in (C) shows that the flow jets extended below the narrow channels connecting the intracranial and cervical SAS and in this region vortices were formed.

Fig 4A, 4B and 4C displays streamlines at the time of peak systolic inflow ($t_{Q_{CS_{\text{max}}}}$) in Con1, P1, and P2, respectively. The velocities increased from FM and caudally in all models. Peak systolic velocities are listed in Table 1. In Con1, the flow was uniform and unidirectional, and maximum velocity in the cervical SAS was 59 mm/s, while the maximum velocity in the aqueduct was 81 mm/s. The time of peak systolic velocities in the cervical SAS did not coincide with peak velocities in the aqueduct. Therefore, there was flow in opposite directions and flow vortices forming in the 4^th^ ventricle.

**Table 1:** Peak systolic velocities at the three given levels and Δ*p* at its maximum, at its minimum, and at the time of peak systolic flow (*Q*_*max*_).

	|u|_*max*_CS	|u|_*max*_Aq	|u|_*max*_FM	$\Delta p_{{\left(PC-CS\right)}_{max}}$	*$\Delta p_{{\left(PC-CS\right)}_{\text{min}}}$*	*$\Delta p_{{\left(Aq-PC\right)}_{\text{max}}}$*	*$\Delta p_{{\left(Aq-PC\right)}_{\text{min}}}$*	*$\Delta p_{{\left(PC-CS\right)}_{t_{Q_{max}}}}$*
	[mm/s]	[mm/s]	[mm/s]	[Pa]	[Pa]	[Pa]	[Pa]	[Pa]
Con1	59	134	27	33.6	-17.4	13.1	-14.4	2.8
P1	43	38	123	44.4	-14.6	1.4	-1.5	4.4
P2	98	116	439	83.9	-39.6	8.2	-6.4	53.6

In the cervical SAS, the maximum velocity for P1 was 43 mm/s and for P2 it was 98 mm/s. At FM, the maximum velocities (123 mm/s) were higher in P1 than in Con1, even though the inflow, $Q_{CS_{max}}$, was lower. The maximum velocity occurred in the narrow passage between the intracranial and cervical SAS, where there was a jet and a tendency to vortex formation. The maximum velocities (439 mm/s) in P2 were almost four times as high as in P1. In the narrow channels connecting the intracranial and cervical SAS flow, jets were forming and these extended below the narrow channels. Vortices formed in the region with larger cross-section areas (Fig 4C). These vortices caused synchronous bidirectional flow.

**Fig 5:**
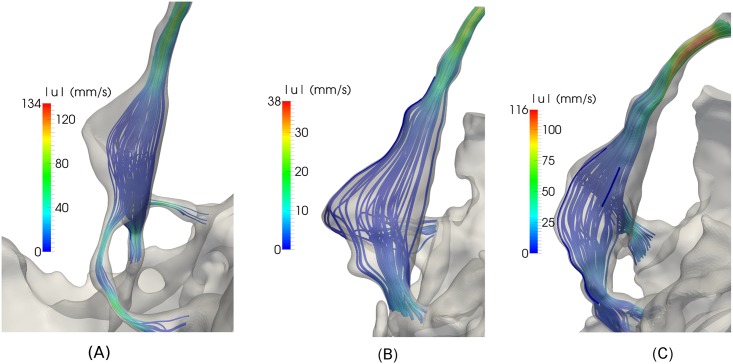
Peak systolic flow in the 4^th^ ventricle in Con1 (A), P1 (B), and P2 (C). The streamlines demonstrate that the flow is uniform and unidirectional. Due to a narrow aqueduct, the maximum velocities were higher in Con1 than P2, although the inflow was higher in the latter. The maximum velocities were low in P1 because of the low inflow rate.

Fig 5 shows peak systolic flow in the 4^th^ ventricle. Due to a narrow aqueduct in Con1, the maximum velocities were 134 mm/s in Con1, compared to only 116 mm/s in P2, despite the fact that the inflow $Q_{Aq_{max}}$ was higher in the latter subject. The maximum velocities were low (38 mm/s) in P1 since the flow rate in the aqueduct was low.

S1 and S2 Animations show the velocity magnitude of Con1 and P2 during the cardiac cycle. P2 demonstrate disturbed flow below FM, while Con1 and P1 appear smooth.

### Pressure and pressure drop

**Fig 6:**
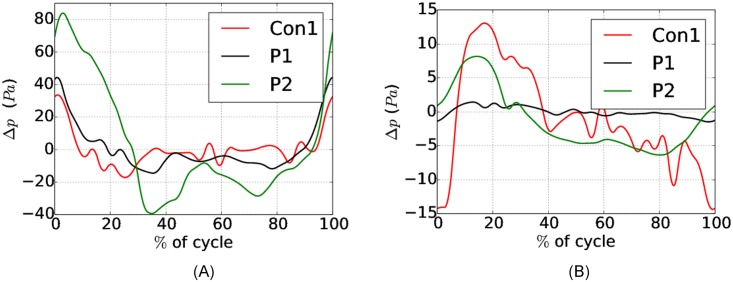
Pressure drop between (A) the cervical SAS (CS) and the pontine cistern (PC) and (B) between the Aqueduct (Aq) and the pontine cistern (PC).

Fig 6a) displays the pressure drop Δ*p*_*PC*−*CS*_ as a function of the time percentage of the cardiac cycle. As mentioned, the pressure at each level represents a spatial average at the given cross-section (the flow was confirmed fully developed in these slices by inspection). The cardiac cycle was defined to start at flow reversal at the cervical inlet/outlet boundary, i.e., t = 0 when Q_CS_ = 0 (see Fig 2).

Maximum Δ*p*_*PC*−*CS*_ was 33.6, 44.4, and 83.9 Pa or 4.5, 5.9 and 11.2 Pa/cm for Con1, P1, and P2 respectively (Table 1), demonstrating that the pressure drop across FM increased with increasing levels of obstruction. At peak systolic flow, Δ*p*_*PC*−*CS*_ was close to zero in Con1 and P1. P2 had $\Delta p_{{\left(PC-CS\right)}_{Q_{\text{max}}}}=53.6$ Pa, which is a factor ten higher than in P1 and P2 (Table 1).

The waveform of Q_CS_ varied between the subjects. Caudal flow lasted 26% of the cycle for Con1, 47% for P1, and 37% for P2. Peak systolic flow was reached after 8, 17.5, and 14% of the cycle for Con1, P1, and P2, respectively. Consequently, the maximum pressure differential did not coincide in the different models.

In Fig 6b), Δ*p*_*PC*−*CS*_ is displayed, and also here we have t = 0 when Q_CS_ = 0. Maximum Δ*p*_*Aq*−*PC*_ was 13.1, 1.4, and 8.2 Pa for Con1, P1, and P2, respectively (Table 1).

Due to the low flow rate Q_Aq_ in P1, the peak of Δ*p*_*Aq*−*PC*_ was low. Con1 had the highest peak Δ*p*_*Aq*−*PC*_, because of the narrow aqueduct, foramen Luschka, and foramen Magendie.

Peak Δ*p*_*PC*−*CS*_ did not coincide with peak Δ*p*_*Aq*−*PC*_, because the flow in the aqueduct (Q_Aq_) was delayed compared to the flow in the SAS (Q_CS_). In Con1,$Q_{Aq_{max}}$ occurred 28% later in the cycle than $Q_{CS_{max}}$, while in P1 and P2, $Q_{Aq_{max}}$ was delayed by 15% and 16%, respectively. The $\Delta p_{{\left(PC-CS\right)}_{\text{max}}}$ quantity coincided with diastolic peak flow $Q_{Aq_{max}}$ in Con1, while systolic peak flow occurred close to Δ*p*_*PC*−*CS*_. For P2, Δ*p*_*PC*−*CS*_ was close to zero at peak systolic and diastolic flow, while Δ*p*_*PC*−*CS*_ peaked at flow reversal, i.e., when Q_Aq_ = 0.

### Hydrodynamical measures

The computed resistances R_PC-CS_ and R_Aq-PC_ in Con1, P1, and P2 are listed in Table 2. (Note that the units are gram · mm^4^ /s). R_PC-CS_ was a factor three higher in P2 compared to Con1, which demonstrates that the resistance increased with increasing levels of obstruction. On the other hand, R_Aq-PC_ was highest in Con1 due to the narrow aqueduct, foramen Luschka and foramen Magendie. In Table 2 also the phase difference θ is listed. The phase difference was smallest in Con1, because the time from zero to peak systolic flow was short.

**Table 2:** Resistance at peak systolic and diastolic flow, and phase difference.

		Con1	P1	P2
$R_{{\left(PC-CS\right)}_{sys}}$	g/(mm^4^s)	0.008	0.018	0.023
$R_{{\left(PC-CS\right)}_{dia}}$	g/(mm^4^s)	0.006	0.006	0.017
$R_{{\left(Aq-PC\right)}_{sys}}$	g/(mm^4^s)	0.072	0.022	0.020
$R_{{\left(Aq-PC\right)}_{dia}}$	g/(mm^4^s)	0.072	0.022	0.020
θ	[-]	0.27	0.36	0.31

## Discussion

In the current study, we used CFD to simulate CSF flow and pressure in the cervical SAS, pontine and cerebellomedullary cisterns, and 4th ventricle in two Chiari patients and one control under subject-specific anatomy and flow conditions. The results demonstrated differences in flow velocities, pressure drop, and flow resistance between the control and the Chiari patients and also between the two Chiari patients. The peak systolic velocity at FM was almost a factor five higher in P1 than in Con1 and a factor 15 higher in P2.

The pressure drop across the FM $\left(\Delta p_{{\left(PC-CS\right)}_{\text{max}}}\right)$ was in the same range for Con1 and P1, while it was a factor two higher in P2. Maximum pressure drop occurred at different times during the cardiac cycle in the different models. The resistance (R_(PC-CS)_) in P2 was slightly higher than in P1, and more than twice the resistance in Con1. In general, the flow was more complex in the Chiari patients with jets forming at peak systolic flow, periods of synchronous bidirectional flow, and reduced phase difference between pressure and flow.

### Comparison to other studies

Ordinary 2D PC-MRI reports peak systolic velocities up to 100–120 mm/s in Chiari I patients [17–24]. On the other hand, 4D PC-MRI studies have reported peak velocities ranging up to 380 mm/s and more complex flow patterns [25]. In the current study, we obtained peak velocities up to 439 mm/s at FM obtained flow jets and vortices similar to those observed in 4D PC-MRI. Especially in P2, the flow patterns in the region of FM were similar to data by Bunck et al. [25].

The peak velocities computed at systolic flow in P2 were high and the assumption of laminar flow may be questionable as also suggested in a recent study by members of our group [8]. Indeed, in a companion study where direct numerical simulations using the Lattice-Boltzman it was found transitional flow in P2 [16]. The observed vortices resulted in synchronous bidirectional flow throughout the flow cycle and not only at the time of flow reversal as recently reported [14]. The pressure drop (maximal systolic pressure drop was 11.2 Pa/cm in P2 from PC to CS) was in the range reported in previous CFD studies [26–29] and comparable to measured values [30,31]. The relation between CSF velocity and pressure is complex. For Con1 and P1, our study confirmed previous studies showing that peak flow coincides (in time) with minimum pressure drop and peak pressure drop with flow reversal [26,27], while in P2 the pressure drop at peak flow ($\Delta p_{t_{Q_{max}}}$) is substantial. A similar phase shift in time has also been reported previously in models with severe obstructions [31] or arachnoiditis [26].

Theoretically, phase shifts of the CSF pressure relative to the arterial blood pressure facilitates flow of CSF into the spinal cord tissue and thereby causes a one way valve mechanism [26]. As mentioned, we observed a phase shift of the pressure relative to unobstructed flow in the presence of a severe stenosis. That is, in our study peak systolic flow occurred later in the cardiac cycle in the Chiari patients than in the control. Delayed peak flow has also been observed in Bunck et al. [25].

The systolic peak velocities in the aqueduct were similar to Kurtcuoglu et al. [32], who modelled pulsatile flow through the 3^rd^ ventricle and aqueduct. Periods of flow in opposite directions in the SAS and 4^th^ ventricle were consistent with Gupta et al. [33], but the velocities were lower in their study.

Under normal flow conditions, CSF flow in the aqueduct (Q_Aq_) is delayed compared to flow in the cervical SAS (Q_CS_). In the PC-MRI data used in the present study, the delay is smaller in the Chiari patients P1 and P2 than in Con1, which implies increased pulse wave velocities and decreased compliance.

### Limitations

There are some limitations with this study caused by uncertainties in the MRI data and underlying assumptions in the CFD model. In the analysis of the PC-MRI data, we had to use an anti-aliasing filter in P2, and in Con1 the low signal to noise ratio made it necessary to define the flow region (ROI) manually. Automatic adjustment and control of V_ENC_ is still not performed at the hospital. Therefore, the filtering and definition of the ROI was performed together with an experienced neuroradiologist (GAR) and found adequate for the study. PC-MRI flow data in the pontine and cerebellomedullary cistern was not obtained and we therefore estimated the flow based on mass conservation. Only Con1 has a flow inlet in the cerebellomedullary cistern, and here the flow rate was scaled by area. The flow is consistent with previous studies, which demonstrate that only around 10% of the CSF flow below FM originates in the ventricular system [34]. On the other hand, the current study may overestimate the flow originating in the cerbellomedullary cistern which was on the order of 30% in our study, but only 10% in Gupta et al. [33]. The study [33] did CFD and measurement of only one individual and there may be variations between individuals. The T2 heavily fluid weighted 3D steady state echo images also varied and was either 0.5 mm x 0.5 mm or 1 mm x 1 mm in the different individuals and the different resolution may have affected the segmentations.

In P2, it could be that some fluid escaped through other routes than the two narrow channels causing an overestimation of the velocities. However, care was taken to represent the underlying anatomical data and the image had clear contrast showing the boundaries between CSF and surroundings.

Our computational model employed rigid walls and enforced mass conservation. However, in Yiallourou et al. [29] it was found that the volume flow was changing along the cervical SAS with peak flow at the level of C2, before it decreased caudally. Change in volume flow is most likely due to structural motion of the surrounding tissue.

As most CFD studies, we neglected fine anatomical structures in the SAS because of the resolution of and noise in the MR data. Yiallourou et al. [29] argued that fine anatomical structures are one of the main reasons why CFD simulations, as this current study, demonstrate high flow rate in the posterior SAS, while 4D PC-MRI clearly shows preferential flow in the anterior SAS [25,29,35]. This is plausible given that 90% of the CSF enters the cervical SAS via the pontine cistern [33,34], and the density of the trabeculae is lower in the anterior than posterior SAS [29]. It is also possible that the nerve roots and dental ligaments limit the flow from the anterior to the posterior SAS. Micro-structures such as nerve roots, denticulate ligaments, and trabeculae have been the subject of several recent studies [36,37] where high resolution geometry resolve the fine structures. It has been found that these microstructures induce vortex formation and mixing and, further, that the pressure may change by a factor as large as 2.5. These microstructures are too small to be reliably identified in a patient-specific manner using MR.

In the current study, the measure of resistance is simply the ratio between maximal pressure drop and velocity. More advanced models of resistance designed for pulsatile flow has been discussed in [38]. In particular, longitudinal impedance provides a measure that takes into account both the tapering of the SAS and pulsatile effects of the flow. While our simple concept of resistance proved sufficient for the analysis in our case, longitudinal impedance may provide a more accurate quantification.

## Conclusions

By incorporating a larger portion of subject-specific anatomy, this CFD study reproduced flow jets and vortices seen in 4D PC-MRI. Peak systolic velocities, phase difference between maximal pressure and flow, and flow resistance distinguished the Chiari patients from the control. Peak pressure drop distinguished the patient with the most severe obstruction from the patient with a moderate obstruction.

## Supporting Information

S1 Animation CSF velocity magnitudes in Con1. (AVI)

S2 Animation CSF velocity magnitudes in P2. (AVI)

## Abbreviations

CSF: Cerebrospinal Fluid
SAS: Subarachnoid space
CS: Cervical SAS
C1: First cervical vertebrae
T1: First thoracic vertebrae
FM: Foramen magnum
Aq: Aqueduct
Con1: Patient with normal anatomy and flow
P1: Chiari patient 1
P2: Chiari patient 2
PC-MRI: Phase Contrast Magnetic Resonance Imaging
V_enc_: Velocity encoding
